# Sea surface circulation in the Baltic Sea: decomposed components and pattern recognition

**DOI:** 10.1038/s41598-024-69463-8

**Published:** 2024-08-12

**Authors:** Amirhossein Barzandeh, Ilja Maljutenko, Sander Rikka, Priidik Lagemaa, Aarne Männik, Rivo Uiboupin, Urmas Raudsepp

**Affiliations:** Department of Marine Systems, Tallinn University of Technology, 12618 Tallinn, Estonia

**Keywords:** Geostrophic current, Ageostrophic current, Baltic Sea, Circulation pattern, Machine learning, Physical oceanography, Atmospheric dynamics

## Abstract

By decomposing the total sea surface current into its geostrophic and ageostrophic components, we examined the contribution of each to the long-term variability of the total sea surface current. Our findings demonstrate the importance of geostrophic currents in Baltic Sea gyre formations. Additionally, ageostrophic currents contribute significantly to the flow across the region. Quantifying the difference between total sea surface current fields has revealed two dominant general sea surface circulation patterns in the Baltic Sea, whose characteristics correspond to the monthly mean climatology of sea surface current fields in May and December. Subsequently, a machine learning technique was employed to effectively detect the types of sea surface circulation patterns using wind vectors and sea level anomaly fields. This underscored the combined influence of sea level anomaly-driven and wind-driven components in shaping surface current vectors in the Baltic Sea, consistent with geostrophic and ageostrophic decompositions.

## Introduction

The surface circulation in the ocean and marginal seas has become an increasing research focus in recent years, driven mainly by the success of satellite remote sensing. Among the dominant components of ocean surface dynamics, the geostrophic and Ekman currents stand out^1–3^. Various datasets, such as Ocean Surface Current Analyses Real-time (OSCAR) and Geostrophic and Ekman Current Observatory (GECKO) products, offer daily maps illustrating these primary currents. Each dataset provides global surface current information derived directly from satellite altimetry and ocean vector winds^4,5^. Their methodologies differ primarily in their handling of geostrophic currents at the equator, addressing wind-driven turbulence, and incorporating adjustments based on sea surface temperature gradients. Despite not encompassing more complex physics such as nonlinearities, these methods stand out due to their minimal assumptions, striving to produce surface current measurements on a consistent global grid at regular intervals that closely resemble direct satellite observations^6–8^. Nonetheless, while these products offer valuable insights into open oceans and some major upwelling zones by emphasizing the role of the wind-driven component in total sea surface current, their estimations may not provide precise details about coastal and marginal seas.

However, validating satellite-based surface currents remains a challenging task due to the scarcity of surface measurements unaffected by surface wind drag on floats^9–11^. Hence, numerical circulation models with acceptable accuracy serve as references for circulation patterns. Additionally, existing knowledge about surface water circulation is invaluable. Understanding the intricate patterns of surface circulation is crucial as a benchmark for remotely sensed circulation data. In our study, we concentrate on a comprehensive analysis of the surface circulation in the Baltic Sea, leveraging its well-studied nature and the reasonably accurate circulation models we rely upon. The central focus of our study is understanding the appearances of geostrophic and ageostrophic circulation. It is essential to note that our analysis of circulation in this study does not involve a comparison or analysis of the circulation derived from satellite remote sensing products.

**Figure 1:**
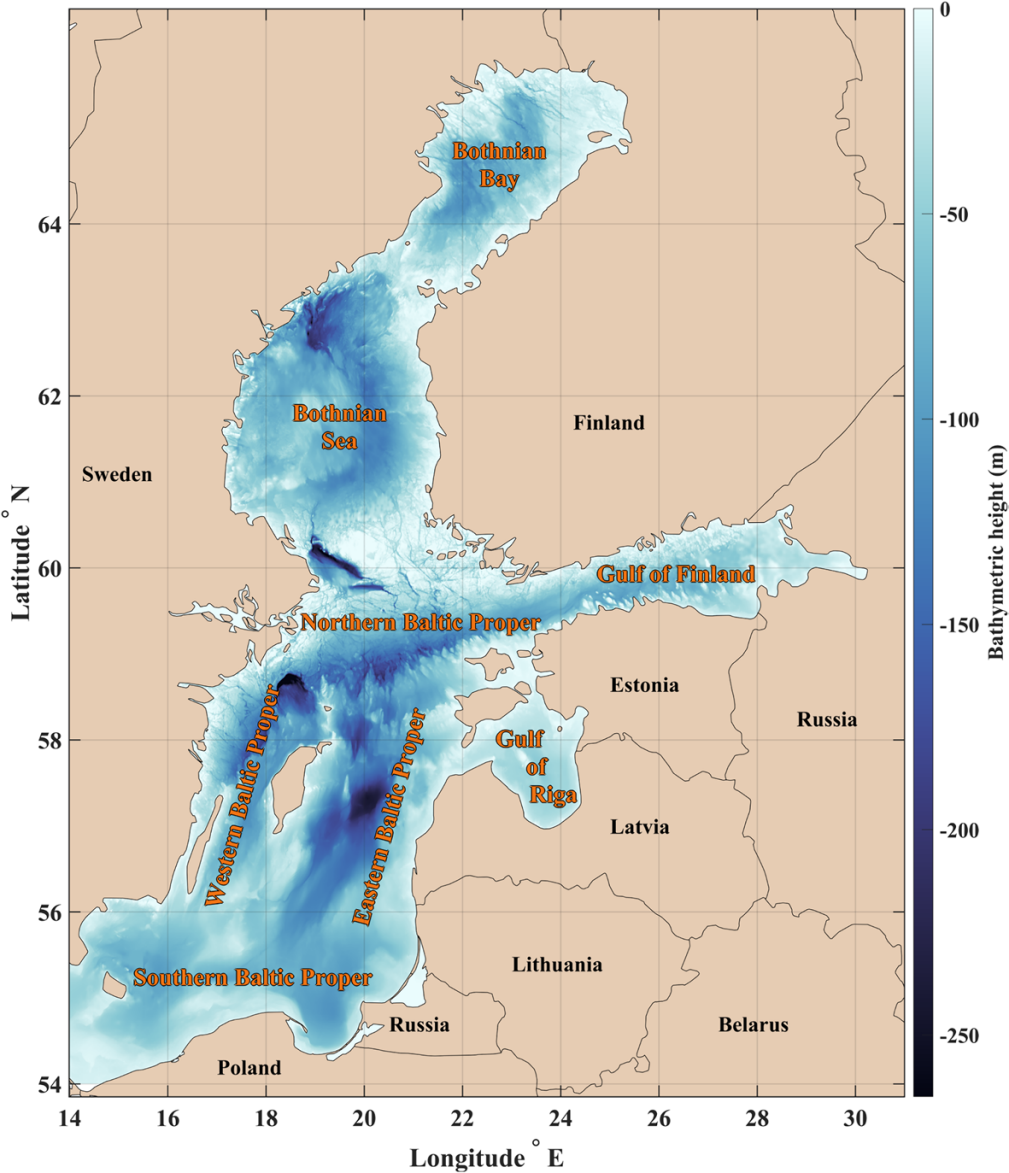
Map of the study area, the Baltic Sea, includes subbasin and country names. The colour scale shows the depth according to the General Bathymetric Chart of the Oceans (GEBCO)^26^ and the final map were generated using MATLAB r2022b programming platform^27^.

The Baltic Sea (Fig. 1) has been extensively studied over the last centuries, leading to the development of relatively accurate circulation models despite its complex arrangement of loosely connected sub-basins. Investigation into the circulation patterns of the Baltic Sea began in the nineteenth century, when Struve (1864) first noted that general currents are caused by a difference in salinity due to the interaction of two distinct water masses within the Baltic basin: the freshwater from extensive runoff and the saline ocean water originating from the North Atlantic Ocean and entering via the Danish Strait^12^. Subsequent studies further substantiated and elaborated upon these initial observations. Sarkisjan et al. were the first to conduct a model-based study that confirmed the presence and significance of cyclonic gyres within the Baltic Sea^13^. Lehmann and Hinrichsen demonstrated that despite significant variability in atmospheric forcing factors, such as wind and sea level pressure, relatively stable annual mean circulation patterns exist in the Baltic Sea, with only occasional inter-annual variations observed in the magnitude of basin-wide cyclonic gyre transports^14^. Omstedt and Axell showed the significant impact of baroclinic flows between the Baltic Sea sub-basins and the Baltic proper while emphasizing the importance of barotropic flow through the Danish Straits and the influence of river runoff^15^. According to Jędrasik et al., surface currents within the Baltic Sea are predominantly influenced by wind dynamics, leading to substantial variability, notably driven by prevailing western winds that produce stronger eastern currents, with an increasing long-term trend in sea surface current intensity^16^. In general, numerous studies employing ocean models have highlighted the prevalence of cyclonic gyres in the overall circulation patterns of both the Baltic Sea as well as its sub-basins^17–23^. In addition, Maljutenko and Raudsepp, and Soosaar et al. illustrated that the general circulation in the Gulf of Finland and the Gulf of Riga can be anticyclonic during spring and summer due to seasonal shifts in the forces driving the sea surface currents^24,25^. Understanding these variations necessitates an assessment of both barotropic and baroclinic perspectives, given their reliance on distinct atmospheric conditions.

Our objective is to analyze the monthly climatology of surface circulation in the Baltic Sea, identifying the dominant spatial patterns of sea surface geostrophic and ageostrophic current components over an annual cycle. Therefore, we aim to decompose the total sea surface current into geostrophic and ageostrophic components. Geostrophic currents are derived from gridded sea surface height anomaly data by calculating the sea level slope. Ageostrophic currents, which can be amplified by various factors, are primarily driven by wind or other non-linear interactions^28,29^. While ageostrophic currents globally may include inertial currents, tidal currents, and Stokes drift, in the Baltic Sea, they are predominantly influenced by Coriolis forces, sea surface topography, and friction, with other factors being negligible^30^. This suggests that geostrophic and wind-driven currents are more significant in this region. However, the combined impact of geostrophic and ageostrophic components on the overall sea surface circulation in the Baltic Sea is still unclear.

The influence of geostrophic and ageostrophic currents on total circulation can change over time due to various forcing factors. This study will also explore the origins of ageostrophic components in the Baltic Sea and their comparison with the geostrophic component caused by sea level anomalies. Our ultimate goal is to categorize each monthly mean circulation pattern into clusters representing dominant circulation patterns. We plan to employ machine learning to determine the dominant type of monthly circulation based on sea surface anomalies and wind fields. Additional spatial and temporal analyses will offer further interpretive insights into the dynamics of sea surface circulation in the Baltic Sea.

## Data and methods

### Data product description and geostrophic/ageostrophic decomposition

The sea level height (*h*) and total sea surface current data (*u*_*tot*_, *v*_*tot*_) for the Baltic Sea (lon: 14 °E to ~ 31 °E and lat: 54 °N to ~ 66 °N) were extracted from the Copernicus Marine Service (CMS) Baltic Sea Physics Reanalysis from January 1993 to December 2021 with a spatial resolution of 1 nautical mile. This data has been produced by the NEMO ocean model of a North Sea and Baltic Sea regional configuration Nemo-Nordic 2.0^31,32^ and it is accompanied by a Quality Information Document (QUID), where it has been reported that the mean correlation between the available tide gauges and CMS Baltic Sea Physical Reanalysis Sea level outputs for the entire Baltic Sea is 0.88 with a root mean square deviation (RMSD) of 0.136 m^33^. The geostrophic velocity components (*u*_*geo*_, *v*_*geo*_) of sea surface current have been calculated from the sea level data as follows^34^:

1a $${u}_{geo}=\frac{-g}{f}\frac{\partial h}{\partial y},$$

1b $${v}_{geo}=\frac{+g}{f}\frac{\partial h}{\partial x},$$

The Eqs. (1a, 1b) are estimated using the 9-point stencil width classic centred difference method^35^. Then, the ageostrophic components (*u*_*ageo*_, *v*_*ageo*_) of sea surface current were calculated by subtracting the geostrophic components from the total current components:

2a $${u}_{ageo}={u}_{tot}-{u}_{geo},$$

2b $${v}_{ageo}={v}_{tot}-{v}_{geo},$$

The long-term mean and monthly means of total, geostrophic and ageostrophic currents over the study period were calculated. The monthly means of the current anomaly were calculated by subtracting the respective monthly mean from the long-term mean calculated over the period of 1993–2021.

### Euclidean distance

The Euclidean distance (ED) was implemented as a useful criterion for determining a single index for assessing the difference between two gridded sea surface current vector fields as below:

3 $$ED=\frac{1}{n\times m}\left({\sum }_{i=1}^{n}{\sum }_{j=1}^{m}\sqrt{{\left({u}_{i,j}^{A}-{u}_{i,j}^{B}\right)}^{2}+{\left({v}_{i,j}^{A}-{v}_{i,j}^{B}\right)}^{2}}\right),$$

where the superscripts *A* and *B* denote two different sea surface current fields, each two-dimensionally gridded along the *x*-axis accounted by subscript *i* with a total number of points *n*, and along the *y*-axis by subscript *j* with a total number of points m.

### Convolutional neural network

A 2-dimensional convolutional neural network (CNN2D) model was trained with monthly mean sea level and wind vector fields to detect the monthly sea surface circulation types (SSCT). The SSCT is defined as a single categorical number representing a specific SSCT. At the preprocessing stage, the wind data from the global atmosphere reanalysis dataset of ERA5^36^ for the period 1993–2021 was extracted over the Baltic Sea area and resampled into the same grids as the CMS data. To feed into CNN2D, each input batch consists of 3 matrices sized 584 × 709, representing monthly mean fields of sea level anomaly (sla), along with zonal (u-wind) and meridional (v-wind) components of 10 m wind speed. The dataset was split into training, validation, and testing sets based on time-based segmentation. The CNN2D was trained on 228 (~ 66%) time-instances of monthly mean fields from 1993 to 2011, validated on 60 (~ 17%) instances from 2012 to 2016, and then tested on the last 60 (~ 17%) instances from 2017 to 2021.

**Figure 2:**
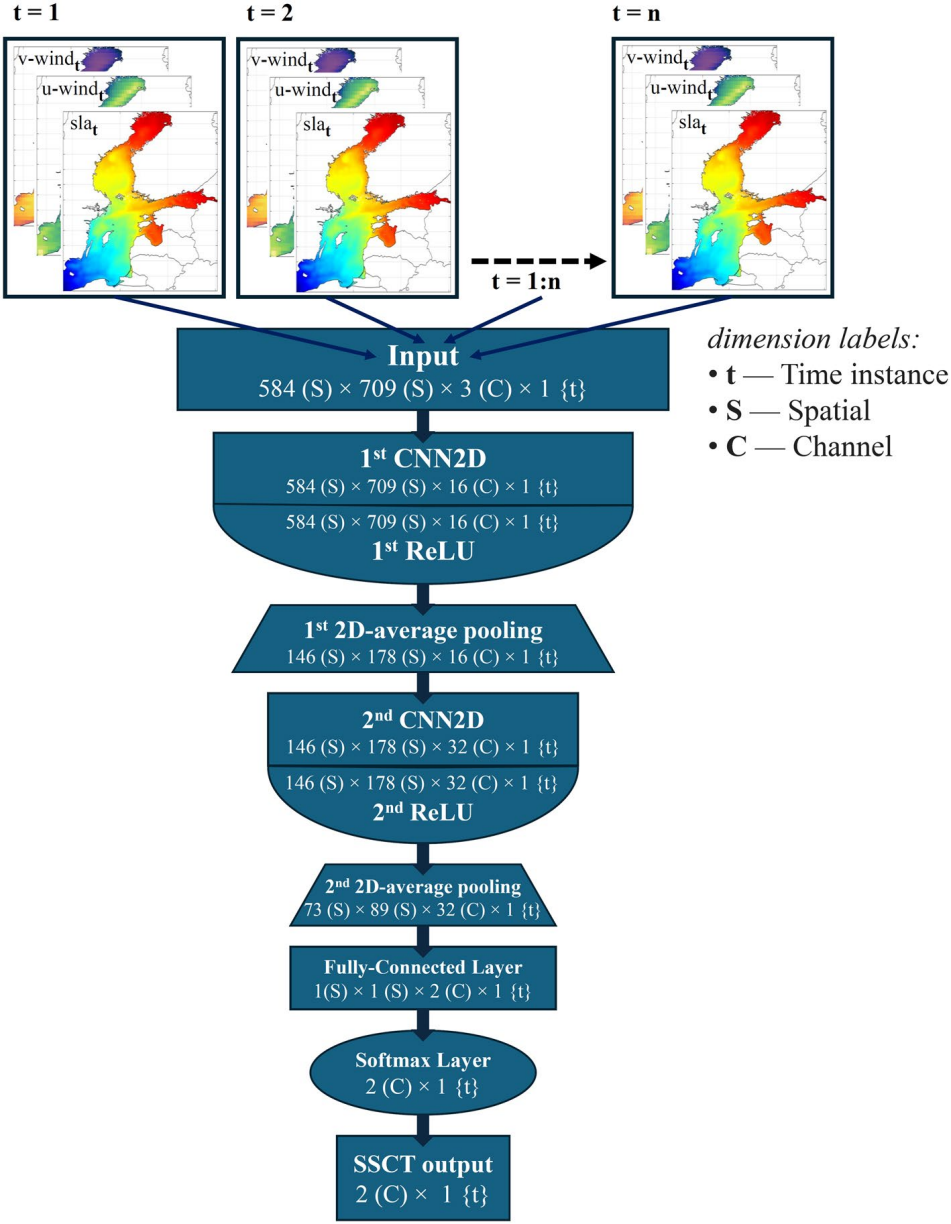
Diagram of the CNN2D model architecture for SSCT recognition from sea level and wind input data.

The architecture of the CNN2D model used for SSCT recognition is presented in Fig. 2. The network starts with an input layer tailored to the size of the training data, followed by convolutional layers designed with filter sizes of 8 × 8 and 16 filters, initially enhancing feature extraction. Rectified Linear Unit (ReLU) activation functions are utilized to introduce non-linearity, followed by average pooling layers of 4 × 4 size to downsample the data^37^. The network further consists of additional convolutional layers with 32 filters with sizes of 4 × 4, followed by similar ReLU activation and average pooling layers of 2 × 2 size. This two-step convolutional approach addresses the large spatial dimensions of the input layers, leading to categorical outputs. A fully connected layer with the number of outputs corresponding to the number of recognized dominant SSCTs precedes the softmax classification layer, facilitating the classification of SSCTs. The network was trained using the 'adam' optimizer with a maximum of 12 epochs, a mini-batch size of 4, an initial learning rate of 0.001, and employed validation data for model evaluation every two epochs^38^.

Furthermore, to gain deeper insights into the contribution of each input feature map—sla, u-wind, and v-wind—in our CNN2D model, we used occlusion sensitivity mapping to analyze the contribution of each input feature in detection of the SSCTs. This involved excluding each feature map from input batches during the testing phase of the trained CNN2D model. Subsequently, we compared the SSCT recognition results with those obtained from the original CNN2D model.

Through these tests, conducted three times with each test excluding one of the maps from the input batches, we evaluated the importance of each component in the CNN2D's decision-making process. Higher error rates observed in these occlusion experiments compared to the original CNN2D results indicate greater dependency of the model on the excluded input component. During the exclusion process, each map—sla, u-wind, and v-wind—was neutralized by filling it with zeros, simulating the absence of data similar to land areas in the data preparation phase. The cumulative number of incorrect SSCT detections, compared to the original labels, was then divided by the total number of time instances to provide the error percentage for the absence of each feature map in the input batches.

## Results

### Mean sea surface current

**Figure 3:**
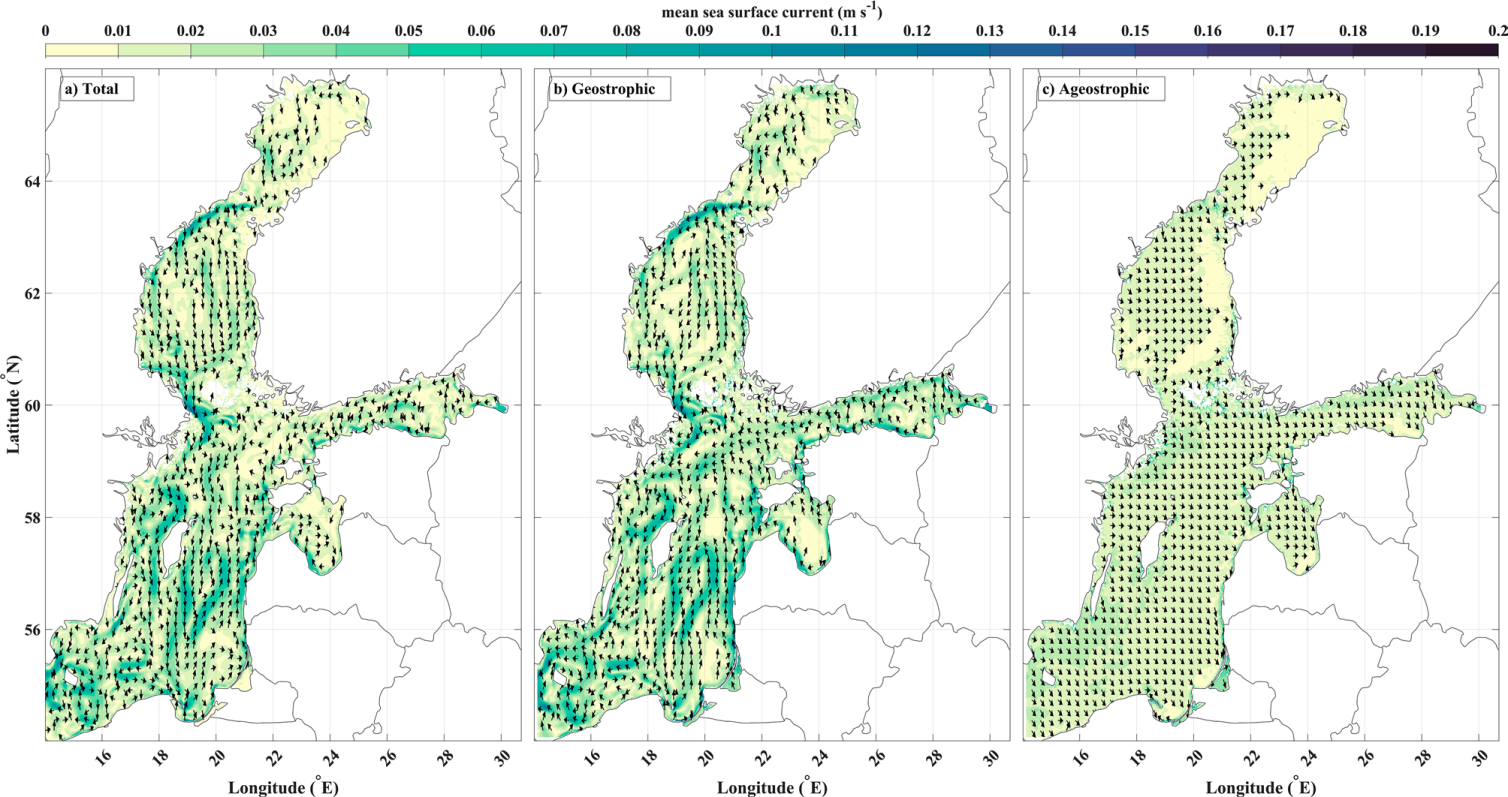
The mean values of (**a**) total, (**b**) geostrophic, and (**c**) ageostrophic surface currents throughout the entire 29-year period (vectors with magnitudes > 0.01 m/s are shown).

Long-term mean sea surface current fields of the Baltic Sea and its decomposition into geostrophic and ageostrophic components are presented in Fig. 3. Geostrophic currents (Fig. 3b) show strong similarity with total currents (Fig. 3a), both in terms of current speeds and patterns. The mean ageostrophic component (Fig. 3c) mainly comprises southeastward currents. Spatial averages are 0.022 m/s for the total currents, 0.025 m/s for the geostrophic currents, and 0.016 m/s for the ageostrophic currents. The geostrophic current primarily represents cyclonic water movement and shapes the dominant gyres (Fig. 3). These gyres exhibit high intensity, particularly around their edges. The weaker ageostrophic currents serve as a uniform background flow almost everywhere.

Ageostrophic currents have a tendency to weaken from the northwestern coast towards the southeastern coasts at the sub-basin scale. Total circulations show prominently cyclonic gyres in the Bothnian Bay, the Bothnian Sea, and the eastern Baltic Proper. Anticyclonic gyres dominate in the Gulf of Riga, the western Baltic Proper, and the eastern part of the Gulf of Finland (Fig. 3a,b). All these patterns are attributed to the geostrophic surface currents, and their magnitude reaches up to ~ 0.15 m/s at the rim of the gyres. The similarity in pattern and strength between total and geostrophic currents indicates that the geostrophic component primarily explains total currents. The ageostrophic component, characterized by a relatively uniform spatial flow direction and lesser speed, contributes secondarily to the total currents.

### Monthly climatology

We present climatological monthly mean circulations for geostrophic, ageostrophic, and total sea surface current. Monthly climatology of geostrophic currents retains its basic spatial structure throughout the year (Supplement, Fig. 2), which is similar to the long-term mean of geostrophic current distribution (Fig. 3b). The ageostrophic component is more variable (Supplement, Fig. 3) in terms of current directions, but the currents still remain directed to the eastward semi-circle in all months (Fig. 3c). The geostrophic currents provide a major contribution to the total currents, which results in the total currents keeping their basic mean structure as well (Supplement, Fig. 1).

**Figure 4:**
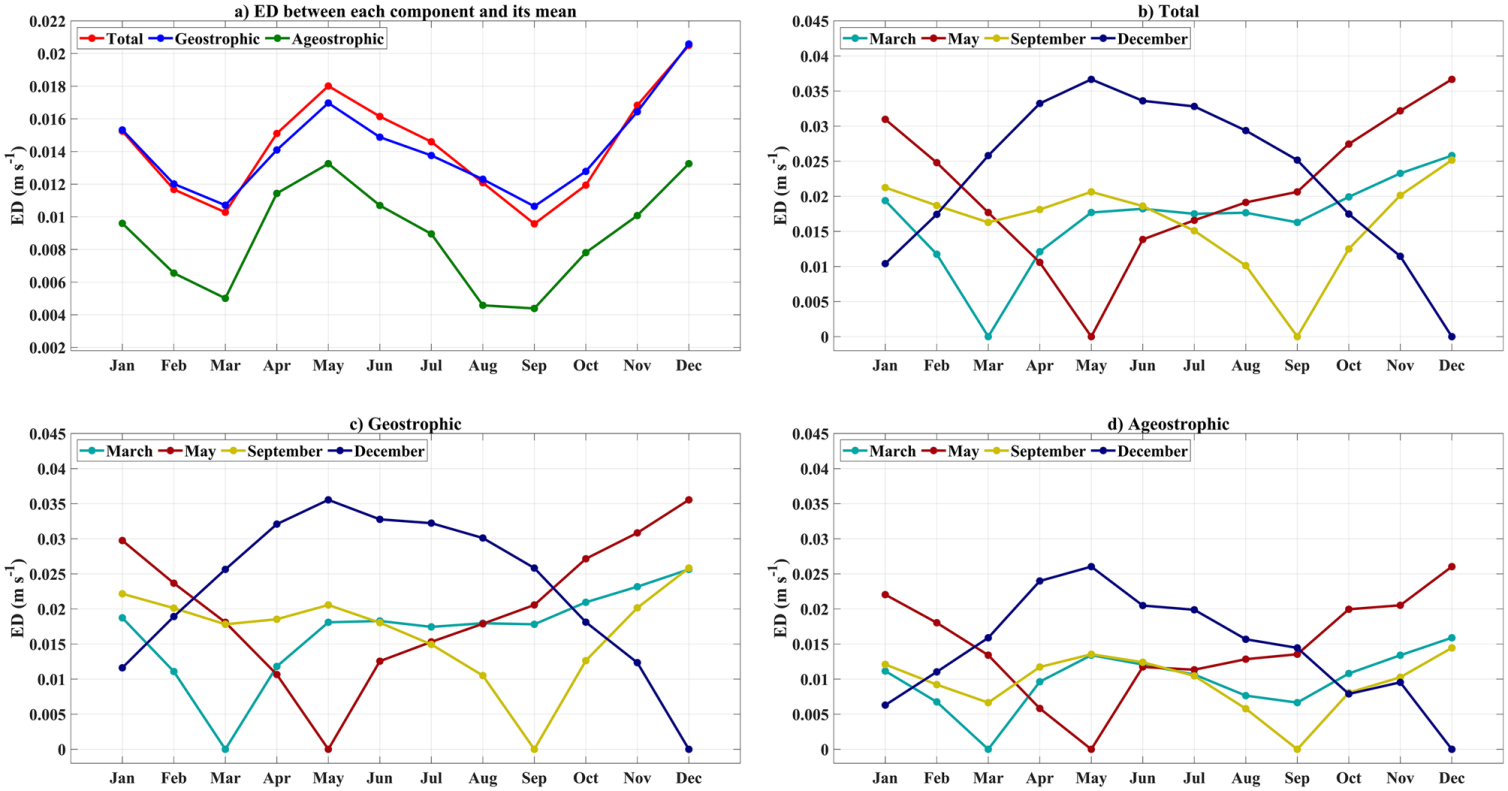
Euclidean distances: (**a**) Euclidean distance between each monthly climatology map and the long-term mean fields. (**b**) Euclidean distance between each monthly climatology map of “total” sea surface current and the mean total current fields in March, May, September, and December. (**c**) Euclidean distance between each monthly climatology map of “geostrophic” current and the mean geostrophic current fields of March, May, September, and December. (**d**) Euclidean distance between each monthly climatology map of “ageostrophic” correct and the mean ageostrophic current fields of March, May, September, and December. A zero value indicates identical circulation patterns. As the ED increases, the disparity between the two circulation patterns also grows.

In order to quantify the disparity between the monthly climatology (Supplement, Figs. 1–3) and the mean maps (Fig. 3), we used ED (Fig. 4a). In this context, the ED reflects the monthly deviation of a particular type of current (total, geostrophic, or ageostrophic) from its long-term mean. The higher the ED value, the greater the difference in the circulation pattern from its reference (Fig. 4).

Figure 4a shows that the SSCTs differ the most from the respective climatological mean in May and December and are most similar in March and September for geostrophic, ageostrophic, and total currents. This means that either the SSCTs in May and December (March and September) are similar to each other or pairwise different. To find out, we conducted an analysis by calculating the ED between each monthly climatological mean circulation and the other 11 monthly means. Our pairwise comparison of monthly climatological circulations revealed that the monthly mean circulations are most closely related to the circulations of the previous and following months (Fig. 4b–d). Notably, there are two significant peaks for each component in May and December, marking the most distinct variation within the year. Furthermore, the SSCTs in April and from June to August exhibit a resemblance to the May SSCT. On the other hand, the SSCTs in October, November, January, and February align closely with the December SSCT. The SSCTs in March and September can be considered as transitional phases, as they separate two distinctive classes (May and December) while being similar to each other.

**Figure 5:**
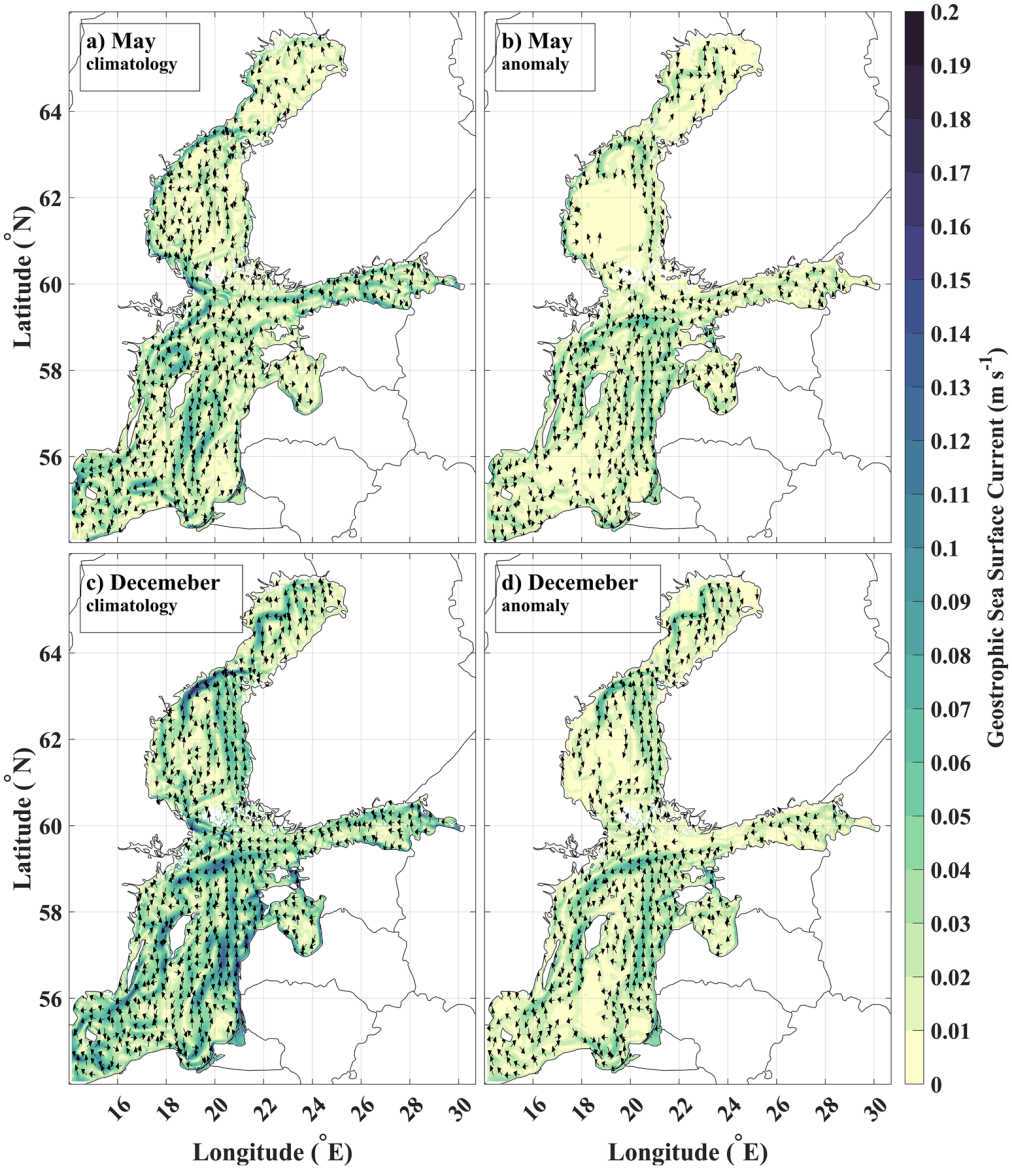
Dominant geostrophic circulation patterns in May and December (vectors with magnitude > 0.01 m/s are shown): (**a**) May climatology. (**b**) May anomaly. (**c**) December climatology (**d**) December anomaly.

**Figure 6:**
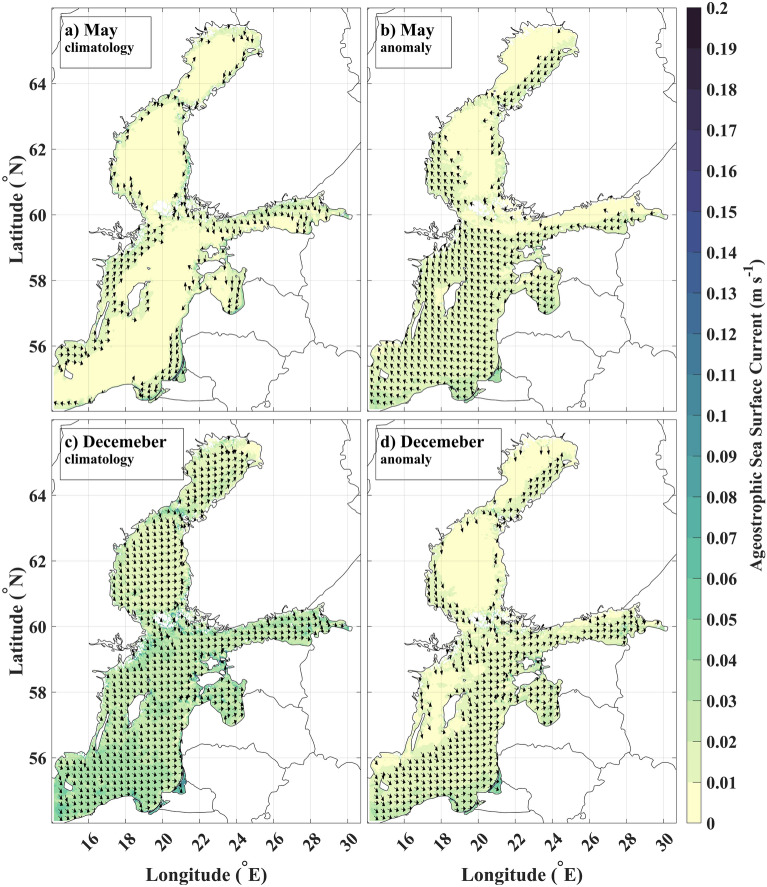
Dominant ageostrophic circulation patterns in May and December (vectors with magnitude > 0.01 m/s are shown): (**a**) May climatology. (**b**) May anomaly. (**c**) December climatology (**d**) December anomaly.

To understand differences between characteristic circulations in May and December, we look at the respective anomalies. Geostrophic circulation in May represents the situation when prevailing cyclonic circulation in the eastern Baltic proper, the Bothnian Sea, and the Bothnian Bay is most distorted (Fig. 5a). Although climatological mean May circulation is still evident, the circulation anomaly shows a dominantly anticyclonic flow scheme (Fig. 5b). The currents in the Gulf of Riga, the Gulf of Finland, and the western Baltic proper are different from the overall anticyclonic tendency. Instead, cyclonic circulation is evident there. The same is true for the circulation in the western Baltic Proper. Predominantly anticyclonic circulation patterns are also present in the anomaly fields of April, June, July, and August (Supplement, Figs. 5 & 6).

Geostrophic circulation in December repeats mean geostrophic circulation in the main circulation directions, but the magnitude of the currents has increased (Fig. 5c). This is well emphasized in the anomaly of the currents in December (Fig. 5d). In most sub-basins of the Baltic Sea, cyclonic gyres dominate. Anticyclonic circulation cells in the western Baltic Proper do not exist in the anomaly field. The separate circulation patterns in the eastern and western Baltic Proper have merged into a single dominant cyclonic gyre that covers the entire Baltic proper. In addition, the anticyclonic circulation in the Gulf of Riga and anticyclonic eddies in the southern Gulf of Finland have disappeared. Predominantly cyclonic circulation patterns are also present in the anomaly fields of October, November, January, and February (Supplement).

Monthly climatological ageostrophic circulation follows qualitatively the same seasonal tendency as geostrophic currents. In May, mean ageostrophic currents exhibit anticyclonic circulation, primarily confined to the coastal areas. The offshore ageostrophic currents are minimal, with speeds less than 0.01 m/s (Fig. 6a). The anomalies of ageostrophic current in May also exhibit anticyclonic shear over the Baltic Sea (Fig. 6b). A similar pattern of ageostrophic currents in the subregions is present in April, June, July, and August (Supplement). Cyclonic shear is dominant from October to February (Supplement), with December being the month most characteristic of cyclonic shear (Fig. 6c,d). The circulation in March and September represents a transition from one SSCT to the other. The currents at that time match the long-term mean patterns the most (Fig. 3c).

### Monthly realizations of SSCTs

**Figure 7:**
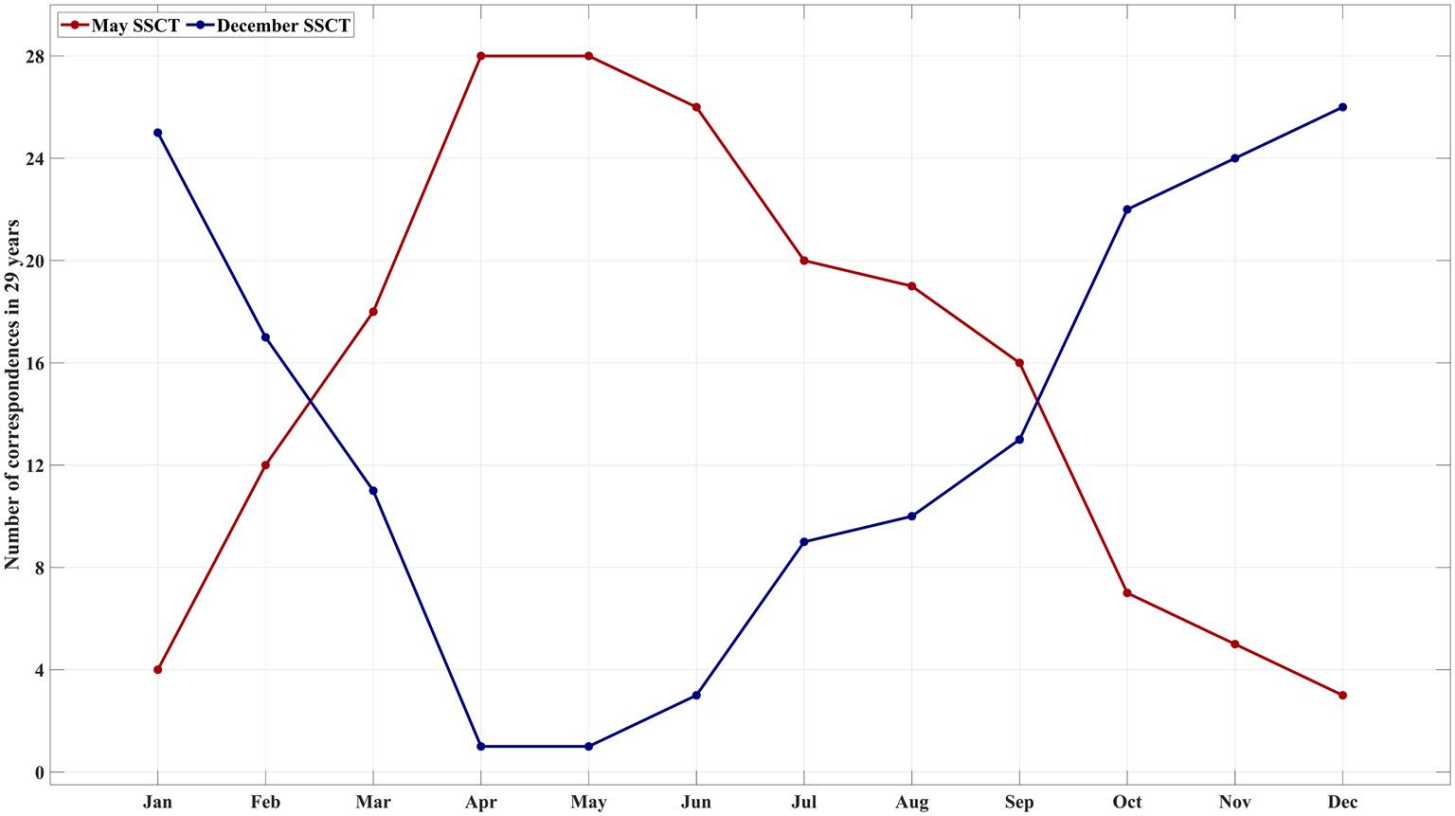
Frequency of SSCT correspondence in each month.

We have identified two primary SSCTs: the strong cyclonically dominated circulation in December represented by climatological December circulation and the weaker one in May represented by climatological May circulation. To measure the monthly prevalence of a dominant SSCT, EDs are calculated pairwise between these SSCTs and all monthly circulations. The smaller of the two EDs each month indicates the presence of the corresponding SSCT for that month. The monthly pattern of this SSCT's dominance supports the seasonality of the characteristic sea surface circulation patterns (Fig. 7). A strong or weak cyclonic circulation is predominant in autumn and winter, or spring and summer, respectively. In transitional months, such as February, March, and September, the similarity to the May or December SSCTs is not clearly defined. During the 29-year study period, there was at least one instance in May or December when the circulation resembled the typical December or May SSCT more closely.

### CNN2D pattern detection

**Figure 8:**
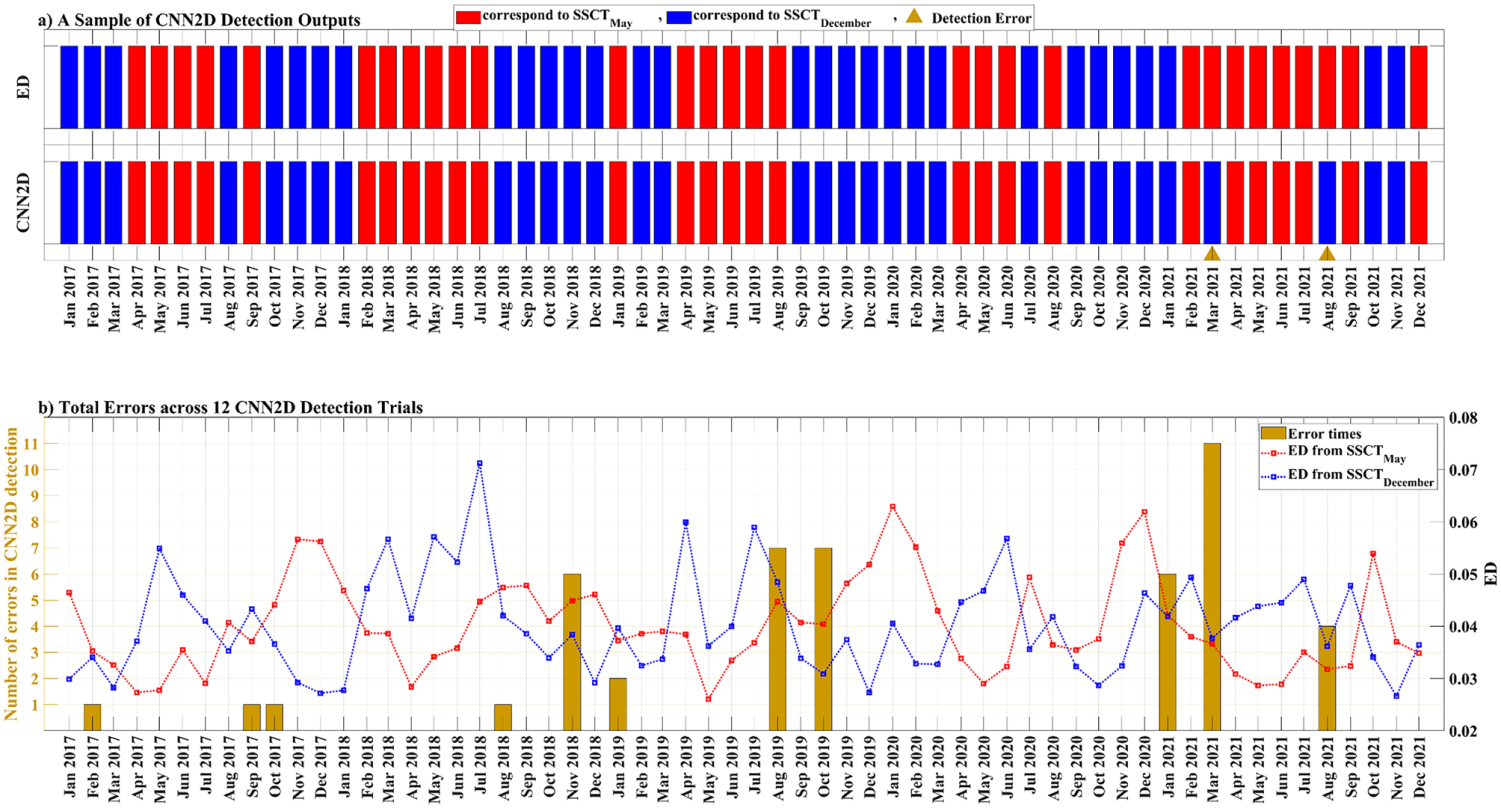
CNN2D results: (**a**) Comparison of the best trial's predicted results with the original SSCT. (**b**) Summary of CNN2D performance across all trials, indicating the number of errors per time instance and the corresponding EDs for each original SSCT.

A CNN2D model was utilized to categorize monthly circulation patterns into two distinct SSCTs that represent the climatological circulations of May and December. The inputs for the model included the monthly mean wind vectors and sea level anomaly fields. The classification results were verified by comparing the SSCTs from the model with the corresponding SSCTs defined by the ED. The smaller ED between the monthly circulation and the climatological circulations of May and December determines the dominant SSCT for each month. Figure 8 depicts the comparison of the model and ED results in identifying SSCTs in the Baltic Sea. The CNN2D model achieved an accuracy of 58 out of 60 in correctly identifying the inherent SSCTs.

Due to the sensitivity of CNN2D, we conducted 12 trials to assess the variability in errors across different training instances. Each trial of CNN2D was conducted with the same configuration and input yet yielded slightly varying outcomes. The most robust trained CNN2D trial resulted in ~ 97% accuracy or 2 errors over 60 testing time instances (Fig. 8a). The range of errors has been changed between different trials from ~ 92% to ~ 97% (from 2 to 5 errors over 60 testing time instances). In addition, the cumulative error temporal variability distribution has been checked for the 12 trials (Fig. 8b). In general, results show that the not-accurate CNN2D detection is at the months where the sea surface current ED from May is close to the sea surface current ED from December. Specifically, errors are most prevalent in March 2021, January 2021, October 2019, August 2019, and November 2018.

Our analysis proceeded with an examination of how sla, u-wind, and v-wind contribute to the decision-making process of our CNN2D model, focusing on the occlusion sensitivity of each map within the input batches. Utilizing our pre-trained CNN2D model, which incorporates all three input fields, we discovered that the omission of sla, while maintaining u-wind and v-wind, led to a 12% error rise in the precise identification of the SSCT. We performed a comparable occlusion sensitivity analysis for both u-wind and v-wind. The results indicate an 8% error increase in identifying the correct SSCT when u-wind data is absent. Conversely, the error rises to 33% when v-wind data is missing.

## Discussion

By decomposing the total sea surface currents into their geostrophic and ageostrophic components, alongside their long-term mean and monthly climatology, our findings underscore the pivotal role of geostrophic currents in forming prevailing gyre structures throughout the Baltic Sea. Accompanying these, the weaker ageostrophic currents provide a consistent background flow across the region. These results strongly imply that the main circulation dynamics in the Baltic Sea are primarily steered by the geostrophic component that is intricately linked to the spatial distribution of static sla. Previous studies have emphasized the significant influence of large-scale wind and air pressure fields on Baltic Sea level variability, with observed correlations with indices such as the North Atlantic Oscillation^39–43^. Indeed, long-term sea surface currents in the Baltic Sea exhibit a direct association with sea level anomalies, which are influenced by wind dynamics, too. Previous studies have considered wind as the primary driver of sea surface currents^23,44^. Our study demonstrates that in the surface layer of the Baltic Sea, geostrophic currents resulting from sea level anomalies can reliably explain the main orientation of sea surface circulations in the Baltic Sea.

Our analysis indicates that ageostrophic currents within the Baltic Sea can be directly driven by wind, as evidenced by their uniform direction with a ~ 90-degree deviation from the prevailing wind direction^45,46^, contributing to an increased anticyclonic tendency in surface circulation patterns. Notably, the intensity of ageostrophic currents decreases from west to east, indicative of the varying influence of wind patterns across the Baltic Sea basin^47^. Our interpretation of the origins of ageostrophic currents in the Baltic Sea is further reinforced by observing their maximum intensity along the Swedish east coast and the Finnish coast^48^. This pattern aligns with the expected dynamics of coastal upwelling events^49,50^, where upwelled waters are horizontally driven towards offshore areas. Furthermore, similar to some previous studies, our analysis has revealed a consistent impact of these ageostrophic components as a share of total sea surface current^51,52^.

Our investigation into the spatio-temporal variability of sea surface currents in the Baltic Sea reveals a predominant categorization into two distinct SSCTs, aligning closely with the mean SSCTs observed in May and December. Moreover, pairwise comparisons between monthly circulation patterns highlight the transitional role of March and September, serving as intermediary phases between the discernible SSCTs identified in May and December. Specifically, the May SSCT is mainly characterized by anticyclonic anomalies, with pronounced differences in circulation patterns across various Baltic Sea subregions. In contrast, December features heightened cyclonic anomalies in the direction of the prevailing circulations of the Baltic Sea. Similar seasonal tendencies are observed in both monthly geostrophic and ageostrophic current distributions and their anomalies, reinforcing the seasonal dominance of SSCT in the Baltic Sea. The frequency distribution of dominant SSCTs underscores the prevalence of strong circulations around autumn and weak circulations around spring, with transitional months exhibiting variability in SSCT dominance.

Despite variations in the intensity of the currents between May and December SSCTs, their directional patterns remain consistent, aligning with the semi-persistence of sea surface currents in the Baltic Sea^53^. Cyclonic circulation predominates in the Bothnian Bay and Bothnian Sea, while anticyclonic circulation characterizes the Gulf of Finland and the Gulf of Riga. However, notable differences in gyre formations persist within the Baltic proper. In the December SSCT, intensified currents extend from the southern Baltic Proper to the northern Baltic Proper through the eastern Baltic Proper. These currents merge with the outflow from the Gulf of Finland and Bothnian Sea before sharply turning southward in the western Baltic Proper (west of Gotland Island), forming a larger cyclonic gyre that covers the whole Baltic Proper. Conversely, in the May SSCT, where currents are weaker, the northward flow from the southern Baltic Proper turns southward along the eastern coast of Gotland Island, forming a cyclonic gyre in the eastern Baltic Proper. Simultaneously, dynamic interactions from the northern Baltic Proper induce an anticyclonic gyre in the western Baltic Proper. This results in the dominance of the May SSCT being characterized by a sea surface gyre dipole in the eastern and western Baltic Proper, a feature absent in the December SSCT. This finding aligns with previous studies that have examined the seasonal variability of the Baltic Sea inflow and outflow^23,54^. Generally, the Baltic Sea inflows/outflows cause high/low sea level anomalies and large-scale meridional gradients in the warm/cold months^55–57^ that support weak and strong geostrophic currents. Also, high/low intensity of wind in the cold/warm months^58,59^ aligns with the strong/weak ageostrophic currents. In fact, the effect of inflow and outflow on sea level and wind distribution refers to how their intensifying and weakening can form different SSCTs in different seasons. While earlier research typically concentrated on water transport across various layers^60,61^, our study has uncovered evidence suggesting that the integration of stronger inflows during colder months into surface layers consolidates into the eastern Baltic currents, reinforcing the December SSCT. By contrast, in the May SSCT, the strengthening of outflows weakens general cyclonic circulation of the Baltic Sea and lays a groundwork for the formation of anticyclonic circulation structures. This nuanced understanding underscores the dynamic interplay between seasonal inflows, outflows, and surface currents, enriching our comprehension of the complex hydrodynamics within the Baltic Sea.

The dominant SSCTs in the Baltic Sea exhibit a sophisticated nature for categorizing the sea surface circulation, especially during the transition months. This implies that being close to May or December in temporal terms does not guarantee an accurate prediction of the sea surface circulation pattern in the Baltic Sea. The results of the present study have shown that the ED can be an efficient and robust metric for SSCT classification in the Baltic Sea. Furthermore, the 2D convolutional neural network model is used for the feasibility study to detect the SSCT with only the potential drivers of wind and sla fields. However, due to the insights gained from the decomposition of total sea surface currents and the uncertainty regarding the origin of the ageostrophic component, the decision was made to exclude other oceanographic quantities or parameters, such as temperature and salinity^62^. This exclusion was deliberate, aimed at preventing any potential bias or artificial improvement in the network's predictive accuracy. Therefore, the constructed CNN2D architecture consists of several layers aimed at processing the spatio-temporal data of wind vectors and sla matrices in the Baltic Sea, accompanied by the fact that both geostrophic and ageostrophic components have important roles in the formation of total sea surface currents in the Baltic Sea.

The CNN2D outcomes demonstrate promising results for SSCT recognition, achieving high accuracy. However, it remains sensitive across different training trials for the network. This sensitivity arises from the complex interactions within the neural network during the training process, leading to different weight adjustments and, consequently, divergent classification results^63^. Notably, the accuracy across the twelve CNN2D trials fluctuates between 2 and 5 errors each time among the 60 time-instances tested. The summation of errors across our twelve trials highlights that the discrepancies correspond to the time-instances when the ED of the sea surface current vector fields for both May and December SSCTs are close to each other. So, it can be concluded that if the wind and sea level distributions in the Baltic Sea are significantly different from the monthly mean climatology distributions, they can cause unusual patterns in the SSCT, which cannot be well categorized into the dominant SSCTs.

Our occlusion experiments revealed that neutralizing sla values while retaining u-wind and v-wind in our pre-trained CNN2D model resulted in a notable 12% increase in error for SSCT detection. Conversely, when either u-wind or v-wind was individually neutralized from the input batches, we observed significant variations in error rates, particularly concerning the meridional component of wind speed. Neutralizing u-wind led to an 8% increase in errors, whereas neutralizing v-wind resulted in a substantial 33% increase in errors. These findings underscore the critical role of wind-driven currents in shaping the different SSCTs.

It's important to note that our pre-trained model, utilizing all three inputs, aims to capture the spatial variability essential for SSCT recognition. However, it is well known that the spatial variability of these quantities, including sla and wind speed, in the Baltic Sea also exhibits a strong seasonal pattern^64–66^. Therefore, these occlusion experiments results are not in conflict with our previous statements regarding the predominant impact of geostrophic currents over ageostrophic currents in the Baltic Sea. This could be the result of seasonal variations in the values of these three inputs, which correspond in such a way that they can partially compensate for the absence of each other. However, they clearly underscore the significance of wind-driven currents in SSCT recognition.

Furthermore, we showed that the higher importance was assigned to the meridional component of winds compared to the zonal component. Previously, it has also been reported that changes in meridional winds were consistent with changes in upwelling frequency, further highlighting the importance of this wind component^46,67^, emphasizing the significant influence of the north–south directionality of wind on Baltic Sea surface dynamics. This also corresponds with the ageostrophic current directional tendencies resulting in the present study. Given the dominance of southeasterly wind in the Baltic Sea, as well as the Ekman theory for deviation of potential wind-driven currents from wind direction, when zonal wind components dominate in the Baltic Sea, then wind-driven currents tend to be more south-ward than east-ward. However, our results for the monthly climatology of ageostrophic currents, as well as the long-term mean of them, showed an eastward tendency of ageostrophic currents in comparison to southward tendencies. This tendency is more observable in the northern sections of the Baltic Sea. This supports the conclusion that wind-driven currents play an important role in SSCTs within the Baltic Sea, with the north–south directionality of wind exerting more influence on circulation patterns.

Eventually, the high accuracy of our main trained model with the full set of inputs, along with the results from our occlusion experiments, supports the hypothesis that, particularly over long-term timescales like monthly averages and beyond, the sea surface ageostrophic component is primarily influenced by wind, with other expected factors being negligible in the ageostrophic stability of the Baltic Sea. In fact, these results suggest that the sea surface circulation fields in the Baltic Sea are predictable well with an AI model which is trained solely on wind and sea level data. Here, the gridded reanalysis database is given an initial insight in this regard. However, remote sensing datasets for both wind and sea level anomaly with reasonable accuracy and spatial resolution may have wonderful potential for the estimation of the sea surface currents in the ocean as well as in coastal and marginal seas^68^. This insight can be useful for conducting future research into the importance of the application of AI in the Baltic Sea surface current and sea level dynamics.

### Supplementary Information

Supplementary Figures.

## Data Availability

The initial datasets utilized in this study are publicly available and can be accessed from the following sources: 1—The sea level anomaly and total surface current from Baltic Sea Physics Reanalysis data are available at Copernicus Marine Service (http://marine.copernicus.eu/). 2—ERA5 wind data are available at Climate Data Store, Copernicus Climate Change Service (https://cds.climate.copernicus.eu/). All other datasets presented in this study are outcomes derived from the procedures outlined in the Data and methods section, and they can be replicated using the aforementioned data and the described methods in the manuscript. Additionally, all datasets generated in this study are available from the corresponding author upon request.
