# Supplementary material for: Sea surface circulation in the Baltic Sea: decomposed components and pattern recognition
Source: Sci Rep. 2024 Aug 12;14:18649. doi: 10.1038/s41598-024-69463-8 (PMC11319359; doi:10.1038/s41598-024-69463-8)
Supplement: Supplementary file 1 — Supplementary Figures. [file 41598_2024_69463_MOESM1_ESM.docx]

**Sea Surface Circulation in the Baltic Sea: Decomposed Components and Pattern Recognition**

Amirhossein Barzandeh^1,*^, Ilja Maljutenko^1^, Sander Rikka^1^, Priidik Lagemaa^1^, Aarne Männik^1^, Rivo Uiboupin^1^, Urmas Raudsepp^1^

^1^Department of Marine Systems, Tallinn University of Technology, Tallinn, 12618, Estonia

*^*^amirhossein.barzandeh@taltech.ee*

**Supplementary Materials**

Supplementary Figure 1, monthly climatology of total sea surface current

Supplementary Figure 2, monthly climatology of geostrophic sea surface current

Supplementary Figure 3, monthly climatology of ageostrophic sea surface current

Supplementary Figure 4, monthly climatology of total sea surface current anomalies

Supplementary Figure 5, monthly climatology of geostrophic sea surface current anomalies

Supplementary Figure 6, monthly climatology of ageostrophic sea surface current anomaliess
